# Electrocatalytic Conversion of CO_2_ to Formate at Low Overpotential by Electrolyte Engineering in Model Molecular Catalysis

**DOI:** 10.1002/cssc.202201566

**Published:** 2022-11-11

**Authors:** Elli Vichou, Albert Solé‐Daura, Caroline Mellot‐Draznieks, Yun Li, Maria Gomez‐Mingot, Marc Fontecave, Carlos M. Sánchez‐Sánchez

**Affiliations:** ^1^ Laboratoire de Chimie des Processus Biologiques Collège de France UMR 8229 CNRS Sorbonne Université PSL Research University 11 Place Marcelin Berthelot 75005 Paris France; ^2^ CNRS Laboratoire Interfaces et Systèmes Electrochimiques LISE Sorbonne Université UMR 8235 4 Place Jussieu 75005 Paris France

**Keywords:** CO_2_ electroreduction, density functional theory, electrolyte engineering, ionic liquids, molecular catalysis

## Abstract

An electrolyte engineering strategy was developed for CO_2_ reduction into formate with a model molecular catalyst, [Rh(bpy)(Cp*)Cl]Cl, by modifying the solvent (organic or aqueous), the proton source (H_2_O or acetic acid), and the electrode/solution interface with imidazolium‐ and pyrrolidinium‐based ionic liquids (ILs). Experimental and theoretical density functional theory investigations suggested that π^+^‐π interactions between the imidazolium‐based IL cation and the reduced bipyridine ligand of the catalyst improved the efficiency of the CO_2_ reduction reaction (CO_2_RR) by lowering the overpotential, while granting partial suppression of the hydrogen evolution reaction. This allowed tuning the selectivity towards formate, reaching for this catalyst an unprecedented faradaic efficiency (FE_HCOO_−) ≥90 % and energy efficiency of 66 % in acetonitrile solution. For the first time, relevant CO_2_ conversion to formic acid/formate was reached at low overpotential (0.28 V) using a homogeneous catalyst in acidic aqueous solution (pH=3.8). These results open up a new strategy based on electrolyte engineering for enhancing carbon balance in CO_2_RR.

## Graphical abstract

**CO_2_ reduction**: The presence of imidazolium‐based ionic liquids in solution significantly impacts on molecular electrocatalysis selectivity and activity by favoring the CO_2_ reduction reaction vs. hydrogen evolution reaction in both acetonitrile and aqueous solution. The combination of electrolytes [EMIM][BF_4_] and acetic acid amplifies their individual roles by enhancing CO_2_ conversion to formate at very low overpotential (0.28 V) in acidic aqueous solution.

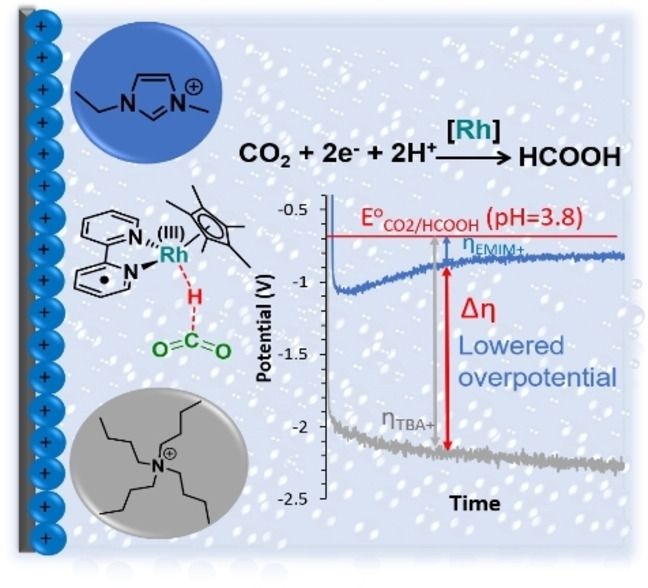

## Introduction

Electrochemical CO_2_ reduction reaction (CO_2_RR) is a promising method for CO_2_ conversion into different value‐added products such as carbon monoxide (CO), formic acid/formate (HCOOH/HCOO^−^), alcohols and hydrocarbons and different heterogeneous and homogeneous catalytic approaches have been already studied.[^1^, ^2^, ^3^, ^4^, ^5^, ^6^] In particular, the production of formate from CO_2_RR is a promising strategy,^[7]^ since formate is a commodity chemical. Molecular catalysis is an interesting approach for CO_2_RR since this type of catalysts offer a high degree of tunability of both the metal center and the ligand.[^8^, ^9^, ^10^, ^11^] However, molecular catalysts are very seldom soluble in aqueous solution,[^12^, ^13^, ^14^] the solvent of choice for industrial applications, and for this reason, most studies are limited to organic solvents. In addition, since protons are required for the CO_2_RR,[^15^, ^16^] hydrogen evolution reaction (HER) represents a critical competitive reaction.

**Figure 1:**
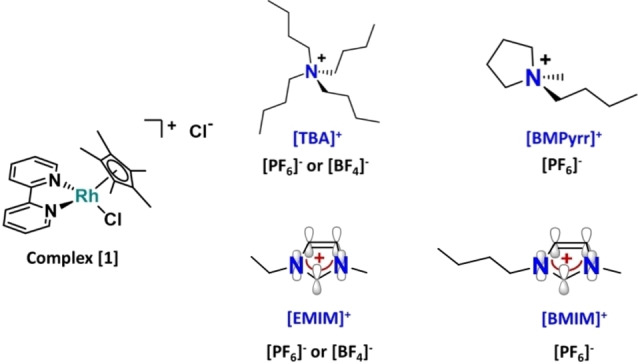
Structures of the [Rh(bpy)(Cp*)Cl]Cl complex [1], where (bpy)=2,2’‐bipyridine and (Cp*)=pentamethylcyclopentadienyl, the benchmark electrolyte [TBA][PF_6_] or [TBA][BF_4_] (tetrabutyl ammonium hexafluorophosphate or tetrafluoroborate, respectively) and all ILs tested: [ΕΜΙΜ][PF_6_]=(1‐ethyl‐3‐methylimidazolium hexafluorophosphate), [ΕΜΙΜ][BF_4_]=1‐ethyl‐3‐methylimidazolium tetrafluoroborate, [ΒΜΙΜ][PF_6_]=1‐butyl‐3‐methylimidazolium hexafluorophosphate, [ΒΜΡyrr][PF_6_]=1‐butyl‐1‐methylpyrrolidinium hexafluorophosphate. An additional schematic representation of the π orbitals is present in the imidazolium‐based ILs.

The molecular catalyst for CO_2_RR is dissolved in the solvent together with the electrolyte. Thus, CO_2_ is not reacting at the electrode surface. In contrast, the molecular catalyst comes into contact with the electrode for a successful electron transfer, which generates the active form of the catalyst, regardless of the chemical nature of the solid electrode used for that purpose. An alternative strategy to improve molecular catalysts’ performances other than the modification of either their metal center or ligands is the modulation of the local electric field by electrolyte engineering using ionic liquids (ILs), since the local environment at the double layer is controlled by the electrolyte composition, but might evolve under operating conditions. So far, most attention has been focused on modulating catalytic electrodes such as Ag or Cu by incorporating ILs, acting as a solvent or a supporting electrolyte[^17^, ^18^, ^19^, ^20^] to influence the catalytic performance (activity[^21^, ^22^, ^23^, ^24^, ^25^] and selectivity^[26]^) of different electrocatalysts,[^27^, ^28^, ^29^] as well as a part of the electrolyte membrane.^[30]^ Thus, such an electrolyte engineering strategy[^31^, ^32^, ^33^, ^34^] aims at controlling the ions present at the electrode–electrolyte interface when the electrical double layer is built up, since it is known to impact the selectivity and the energy efficiency of CO_2_RR. Two main approaches are considered in the literature for that purpose. On the one hand, increasing the hydrophobicity of the electrode surface by addition of long‐chain cationic surfactants such as hexadecyl trimethylammonium bromide (CTAB) in solution or drop casting hydrophobic polymers such as poly(vinylidene difluoride) (PVDF) on the electrode surface, which promotes in both cases HER suppression[^35^, ^36^, ^37^] by forming a nonpolar layer on the electrode. On the other hand, modulating the electric field on the electrode–solution interface, which either stabilizes or destabilizes CO_2_ reaction intermediates.[^38^, ^39^, ^40^] In particular, the potential‐dependent orientation of the ions in the electrical double layer implies that mainly cation adsorption happens at the interface when the electrode undergoes cathodic polarization and anion adsorption under anodic polarization.^[41]^ In contrast, very few studies using ILs in solution have been devoted to molecular catalytic systems.[^42^, ^43^, ^44^, ^45^] In one of those rare examples, we have already demonstrated that ILs in solution acted as catalytic promoters for CO production by decreasing the reaction overpotential, but not affecting the CO_2_RR selectivity.^[43]^ However, the main goal of the present work is to study the effect of ILs not only on the activity, but also on the selectivity (CO_2_RR vs. HER). For that purpose, we used a model molecular catalyst with a well‐established mechanism[^46^, ^47^] for formate production (Scheme S1), [Rh(bpy)(Cp*)Cl]Cl (bpy=bipyridine and Cp*=pentamethylcyclopentadienyl), referred to here as complex [1] (Figure 1). This water‐soluble catalyst exhibits moderate selectivity for CO_2_ conversion to formate (faradaic efficiency FE_HCOO_− ≤50 %) and also presents significant activity as a HER catalyst. Thus, complex [1] represents a suitable model for studying the IL impact on the catalyst selectivity.

Herein, the impact of two different molecular solvents (acetonitrile and water), two types of ILs (pyrrolidinium‐ and imidazolium‐based ILs, represented in Figure 1), and two proton sources (water and acetic acid), on the selectivity and energy efficiency of CO_2_RR displayed by the selected model molecular catalyst (complex [1]) has been evaluated. We show that, thanks to an IL‐based electrolyte, the catalyst allows greater selectivity for formate and higher energy efficiency not only in acetonitrile but, remarkably, also in purely aqueous acidic conditions, which is a rarely reported performance in the case of a molecular complex. This results in an enhanced carbon balance during CO_2_RR regarding the input CO_2_ thanks to the acidic electrolyte, which significantly reduces the amount of CO_2_ captured as bicarbonate (HCO_3_^−^) and carbonate (CO_3_^2−^) in the bulk solution[^48^, ^49^] by conventional strong alkaline or neutral aqueous solutions. However, the main drawback associated with an acidic aqueous electrolyte for CO_2_RR is the more favorable environment for the competitive HER, which highlights the present need of developing new strategies for suppressing HER, such as the one presented here based on an IL electrolyte. Density functional theory (DFT) calculations provide insights into the influence of the ILs on the electronic structure of the catalyst and on the reaction mechanisms at play in both CO_2_RR and HER.

## Results and Discussion

**Figure 2:**
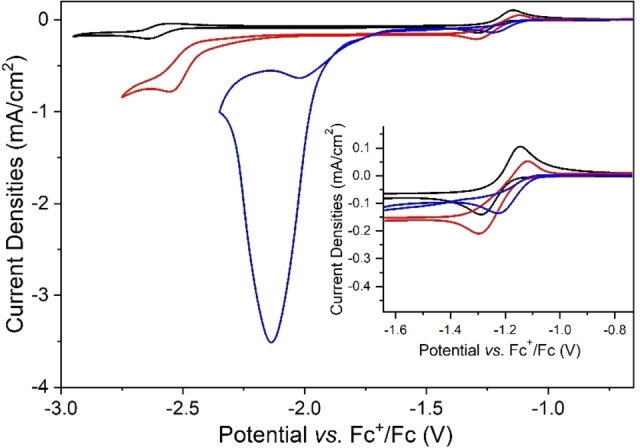
Cyclic voltammograms on glassy carbon (GC) electrode of 1 mm complex [1] and 0.5 m [TBA][PF_6_] in acetonitrile under argon (black plot), under CO_2_ (red plot), and under CO_2_ in the presence of 5 % *v*/*v* H_2_O (blue plot). Inset showing enlarged area on the Rh‐centered redox peak. Scan rate 0.01 V s^−1^.

**Figure 3:**
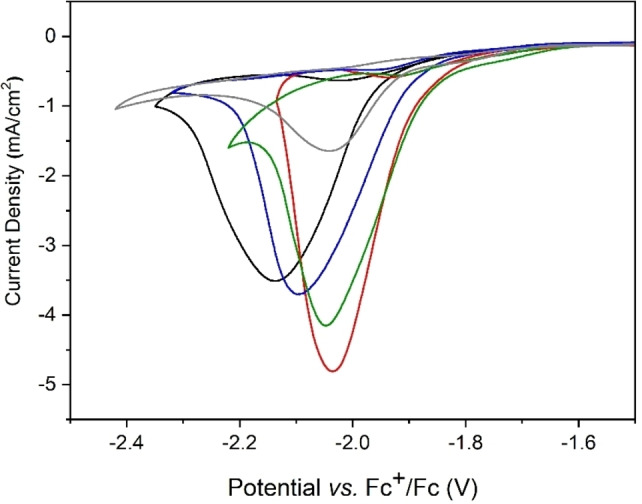
Cyclic voltammograms on GC electrode of 1 mm complex [1] and 0.5 m of different supporting electrolytes in acetonitrile solution containing 5 % *v*/*v* H_2_O under CO_2_. [TBA][PF_6_] (black plot), [BMPyrr][PF_6_] (gray plot), [EMIM][PF_6_] (red plot), [BMIM][PF_6_] (blue plot), [EMIM][BF_4_] (green plot). Scan rate 0.01 V s^−1^.

The electrochemical characterization of complex [1] by cyclic voltammetry in acetonitrile using [TBA][PF_6_] as a conventional supporting electrolyte under inert atmosphere, as well as in the presence of CO_2_, with and without 5 % *v*/*v* H_2_O as a proton source, is reported in Figure 2. Those experimental conditions represent the benchmark conditions previously reported^[46]^ to study electrocatalytic CO_2_ conversion to formate using this model molecular catalyst in organic solvents. According to the literature,[^46^, ^47^, ^50^] the first quasi‐reversible reduction wave observed in the black and red plots represented in Figure 2 and centered at −1.21 V vs. Fc^+^/Fc has been attributed to the metal center reduction from Rh^III^ into Rh^I^. This metal‐centered redox wave is strongly affected by the simultaneous addition of a proton source and CO_2_ in solution (Figure 2, blue plot), whereby it becomes irreversible due to the chemical reoxidation of the catalyst triggered by the catalytic reduction of CO_2_ (see below). In contrast, the second reduction wave, which has been assigned to a one‐electron reduction of the bipyridine ligand shifts from −2.60 V in the absence of CO_2_ and proton source in solution to −2.14 V under those conditions. This is accompanied by a significant increase of current density (*j*_cat_/*j*_p_=17.5, where *j*_cat_ corresponds to the maximum catalytic current density) confirming a catalytic process. Figure S1 shows the effect on the electrochemical response of complex [1] under inert conditions (in the absence of CO_2_ and H_2_O) of the two different types of ILs studied here as supporting electrolyte (pyrrolidinium cation [BMPyrr]^+^, which only contains sp^3^ carbons, and imidazolium‐based ILs [EMIM]^+^ and [ΒΜΙΜ]^+^, which contain sp^2^ carbons) (Figure 1). Moreover, their influence is also evaluated under catalytic conditions, as shown in Figure 3, which compares the benchmark supporting electrolyte and the different types of ILs studied here in a concentration of 0.5 m, since we have already demonstrated in a previous article^[43]^ that no additional effect is provided upon increasing the IL concentration beyond 0.5 m. Moreover, it must be noted that the catalytic current displayed in Figure 3 is independent of the scan rate in all different electrolytes. Table 1 reports the values, determined from Figure 3 and Figure S1, for the following parameters: catalytic potential (potential at the maximal peak current, *E*_cat_), half wave catalytic potential (*E*_cat/2_), catalytic peak current under CO_2_ (*j*_cat_), the ratio of currents under CO_2_ and under argon (*j*_p_) compared at the peak potential value (*j*_cat_/*j*_p_). However, *j*_p_ cannot be observed in some cases because imidazolium cations undergo a reduction process at about −2.5 V, hindering any process occurring at more negative potentials (see Figure S2).[^22^, ^51^, ^52^] The data shown in Figure 3 and Table 1 demonstrate a significant effect of ILs on the catalytic parameters.On the one hand, they all result in lower *E*_cat/2_ as compared to [TBA][PF_6_], the largest decrease (70 mV) being obtained with [EMIM][BF_4_] in solution. On the other hand, ILs have contrasting effects on the maximal catalytic current density displayed, *j*_cat_: [BMPyrr][PF_6_] resulted in decreased current with respect to [TBA][PF_6_], which is probably due to a more hydrophobic character of the electrode–electrolyte interface in the presence of [BMPyrr]^+^, which provokes lower current values. In contrast, all three imidazolium‐based ILs resulted in increased current densities, in the following order: [EMIM][PF_6_]>[EMIM][BF_4_]>[BMIM][PF_6_], which seems to point out that no hydrophobicity modification takes place at the electrode–electrolyte interface. Note that the effect of imidazolium‐based ILs on both the overpotential and the catalytic current density cannot be ascribed to an increase of the local concentration of CO_2_ at the electrode surface, since CO_2_ is less soluble in imidazolium‐based ILs than in acetonitrile^[24]^ and the short‐chain ILs studied do not form any nonpolar layer at the electrode, as surfactants do. Next, we aim to study the CO_2_RR in purely aqueous solution, which remains unaddressed so far for complex [1] despite being a water‐soluble catalyst. In the following, we explore the increase of the amount of water in acetonitrile and finally, the use of purely aqueous solutions at different pH values. For this purpose, and due to the low solubility in aqueous solution provided by electrolytes containing the PF_6_ anion, we chose to compare [EMIM][BF_4_] and [TBA][BF_4_] as electrolytes, since BF_4_ anion exhibits higher solubility in aqueous solution. Thus, two additional solvents were studied: (i) acetonitrile/H_2_O 50 : 50 *v*/*v* (Figure S3) and (ii) H_2_O 100 % solution (Figure S4). Interestingly, we demonstrate that [EMIM][BF_4_] also decreases the *E*_cat/2_ by 70 mV as compared to [TBA][BF_4_] in aqueous solution (Table 1 and Figure S4, blue plots) and furthermore, HER is significantly shifted towards more cathodic potentials, which significantly improves the *j*_cat_/*j*_p_ ratio (Table 1 and Figure S4, black plots).

**Table 1:** Comparison of the catalytic parameters of 1 mm complex [1] and 0.5 m of different supporting electrolytes either in acetonitrile solution containing 5 % *v*/*v* H_2_O under CO_2_ (Figure 3) or aqueous solution under CO_2_ (Figure S4).

Supporting electrolyte	Solvent	*E*_cat_ [V]	*E*_cat/2_ [V]	*j*_cat_ [mA cm^−2^]	*j*_cat_/*j*_p_
[TBA][PF_6_]	CH_3_CN	−2.14^[a]^	−2.00^[a]^	−3.51	17.5
[BMPyrr][PF_6_]	CH_3_CN	−2.04^[a]^	−1.95^[a]^	−1.64	3.10
[BMIM][PF_6_]	CH_3_CN	−2.09^[a]^	−1.97^[a]^	−3.70	–
[EMIM][PF_6_]	CH_3_CN	−2.03^[a]^	−1.94^[a]^	−4.81	–
[EMIM][BF_4_]	CH_3_CN	−2.04^[a]^	−1.93^[a]^	−4.15	–
[TBA][BF_4_]	H_2_O	−1.67^[b]^	−1.45^[b]^	−0.60	0.39
[EMIM][BF_4_]	H_2_O	−1.57^[b]^	−1.38^[b]^	−0.72	3.60

[a] Potentials referred vs. Fc^+^/Fc [V]. [b] Potentials referred vs. Ag/AgCl [V].

**Figure 4:**
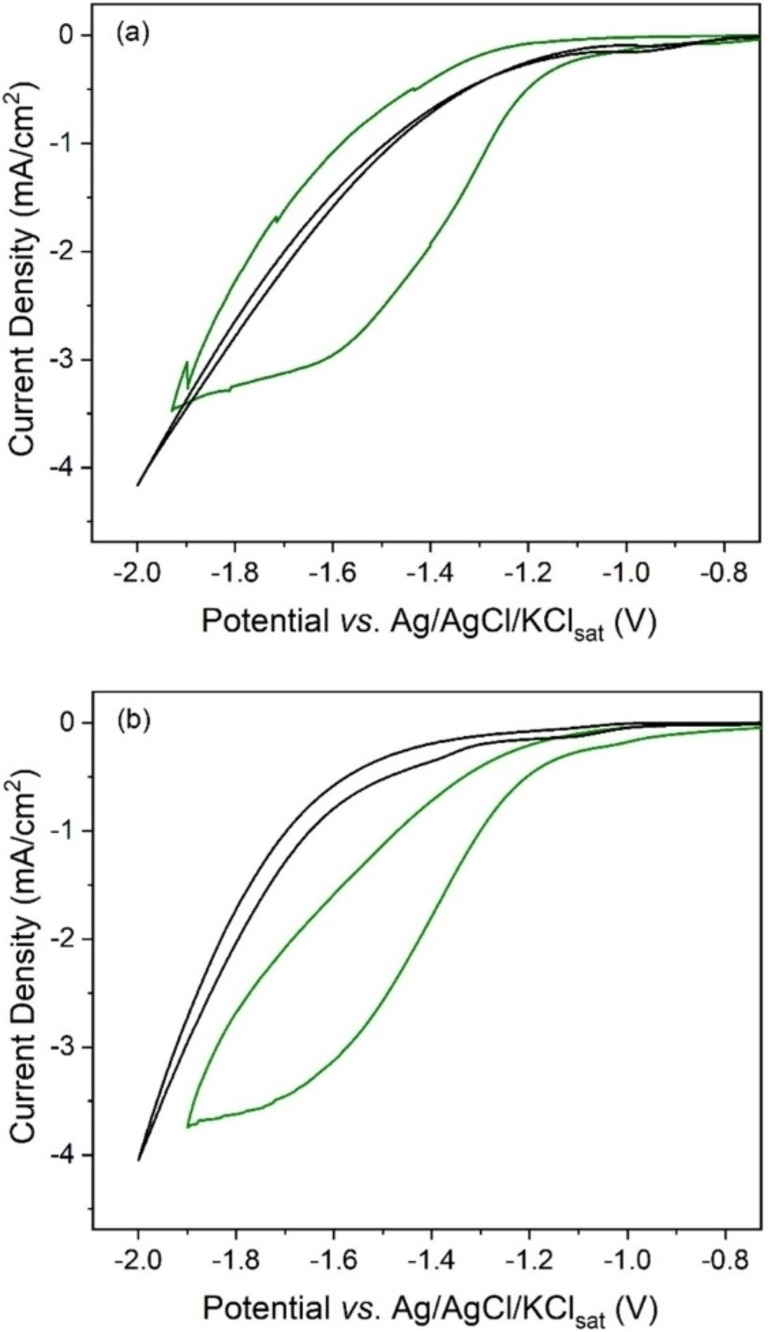
Cyclic voltammograms on GC electrode in an aqueous solution containing: (a) 0.1 m [TBA][BF_4_], 0.1 m acetate buffer (pH=3.8) and 1 mm complex [1] either under argon (black plot) or under CO_2_ (green plot); (b) 0.1 m [EMIM][BF_4_], 0.1 m acetate buffer (pH=3.8) and 1 mm complex [1] either under argon (black plot) or under CO_2_ (green plot). Scan rate=0.01 V s^−1^.

Figure 4 shows the role of adding a weak Brønsted acid (acetic acid) as a more acidic proton donor than H_2_O in solution, together with [TBA]^+^ or [EMIM]^+^. Addition of acetic acid in the presence of CO_2_ (green plots) greatly enhances the catalytic activity of complex [1] with, in both cases, 5 times larger *j*_cat_ value (by comparing Figure 4 and Figure S4). This can be ascribed to a higher concentration of protonated catalyst when reaching *E*_cat_, granted by acetic acid molecules acting as proton donors for direct protonation of the Rh^I^ intermediate (see Scheme S1 for the catalytic cycle). However, acetic acid has almost no effect on *E*_cat/2_ under CO_2_ and [EMIM]^+^ (1.39 V in Figure 4 vs. 1.38 V in Figure S4). Figure 4 (black plots) also shows control experiments in the presence of acetic acid and complex [1], but in the absence of CO_2_, with either [TBA]^+^ or [EMIM]^+^ in solution, which demonstrates a minor contribution from HER catalyzed by complex [1] within the potential range studied herein by electrolysis under acidic aqueous conditions (see Table 3).

The effect on activity, products selectivity, and energy efficiency of the CO_2_RR due to the presence of ILs in acetonitrile and aqueous solution was studied by controlled‐potential (CPE) and controlled‐current (CCE) electrolysis. In all experiments, formic acid/formate was detected as the sole product in the liquid phase, and only H_2_ was observed in the gas phase. Table 2 shows the overpotential, faradaic efficiencies obtained for both products, and energy efficiency for CO_2_ conversion to formate as a function of the electrolyte composition, H_2_O content and either applied potential or current during electrolysis in acetonitrile solution (see Figure S5). As formate can partially migrate from the catholyte to the anolyte,^[7]^ a systematic analysis of both catholyte and anolyte solutions was performed in all electrolysis reported here, proving that between 15 and 20 % of the total formate generated during the electrolysis was detected within the anolyte solution. Thus, analyzing the presence of reaction products in both compartments allowed closing quite efficiently the mass balance of the electrolysis reaching in most cases a total FE (FE${}_{\mathrm{HCOO}{}^{-}}$ +FE${}_{H{}_{2}}$ ) between 78 and 100 %. Table 2 shows that, in all experiments, H_2_ formation is reduced and FE${}_{\mathrm{HCOO}{}^{-}}$ increases upon addition of [EMIM][BF_4_] or [EMIM][PF_6_]. This results in a very selective CO_2_ to formate conversion (FE${}_{\mathrm{HCOO}{}^{-}}$ >90 % and a maximal energy efficiency of 66 %), taking place at much more anodic potentials when compared to [TBA][PF_6_] (Table 2, entries 1–3). These results point out the significant mechanistic role of [EMIM]^+^ cations at the electrode/solution interface in enhancing formate production and partially inhibiting HER. Moreover, the comparison of CCE results reported in Table 2 clearly shows no anion effect neither on activity nor on the selectivity of CO_2_RR, since the overpotential and products distribution remained identical comparing [TBA][PF_6_] and [TBA][BF_4_] as the electrolyte (entries 4 and 5). In contrast, a relevant impact in products selectivity favoring formate production in the presence of [EMIM]^+^ was evidenced, since the products ratio (FE${}_{\mathrm{HCOO}{}^{-}}$ /FE${}_{H{}_{2}}$ ) doubles from (1.5 : 1) to (3 : 1) in comparison with [TBA]^+^ (Table 2, entries 4 and 6). Comparison of the results in entries 4 and 7 of Table 2 allows to rule out any contribution in CO_2_RR from Rh^0^ nanoparticles deposited on the glassy carbon (GC) electrode as a result of complex [1] decomposition/electrodeposition during the electrolysis. For that control experiment, the electrode used during a first electrolysis (entry 4) was recovered, smoothly rinsed and used for a second electrolysis under the same conditions, but in the absence of complex [1], H_2_ being almost the sole product formed in that case (entry 7). This result is very similar to the one obtained with a bare GCE. Then, FE${}_{\mathrm{HCOO}{}^{-}}$ decreased from 53 to 3 % and FE${}_{H{}_{2}}$ shifted from 34 to 94 % by comparing entries 4 and 7. Finally, the comparison of CCE results in entries 5 and 8 of Table 2 reveals a negligible effect in selectivity linked to the % of H_2_O present in solution, since the products ratio (FE${}_{\mathrm{HCOO}{}^{-}}$ /FE${}_{H{}_{2}}$ ) remains almost identical as the H_2_O content in solution increases from 5 to 50 % *v*/*v*. Therefore, this selective conversion of CO_2_ to formate places complex [1] among the top selective molecular catalysts reported in the literature, which exhibit FE${}_{\mathrm{HCOO}{}^{-}}$ =80–97 %.^[14]^ Actually, previously reported results using complex [1] and the benchmark electrolyte ([TBA][PF_6_]) in electrocatalysis[^14^, ^46^] never reached FE${}_{\mathrm{HCOO}{}^{-}}$ ≥50 %.

**Table 2:** Constant‐potential and constant‐current electrolysis in CO_2_‐saturated acetonitrile solution using different electrolytes and % of H_2_O as proton source in an electrochemical two‐compartments H type cell. Total electrolysis duration to circulate 15 C.

Entry	Solvent	Electrolyte	H_2_O [vol. %]	Applied potential [V vs. Fc^+^/Fc]	Applied current [mA cm^−2^]	*E*_cat_^average^ [V vs. Fc^+^/Fc]	*η* ^[a]^ [V]	FE${}_{\mathrm{HCOO}{}^{-}}$ ^[b]^ [%]	FE${}_{H{}_{2}}$ ^[b]^ [%]	Energy efficiency^[c]^ [%]
1	CH_3_CN	[TBA][PF_6_]	5	−2.10	–	–	0.78	77±2	18±5	48
2^[d]^	CH_3_CN	[EMIM][PF_6_]	5	−1.89	–	–	0.57	90±5	9±2	63
3	CH_3_CN	[EMIM][BF_4_]	5	−1.83	–	–	0.51	91±4	9±2	66
4	CH_3_CN	[TBA][PF_6_]	5	–	−3.33	−2.90	1.58	53±2	34±5	24
5	CH_3_CN	[TBA][BF_4_]	5	–	−3.33	−2.90	1.58	56±2	35±2	25
6	CH_3_CN	[EMIM][PF_6_]	5	–	−3.33	−1.95	0.63	69±2	22±3	47
7^[e]^	CH_3_CN	[TBA][PF_6_]	5	–	−3.33	−2.50	1.18	3±1	94±2	2
8	CH_3_CN	[TBA][BF_4_]	50	–	−3.33	−2.60	1.28	47±2	31±1	24

[a] Determined using *E* ^0^ ${}_{\mathrm{CO}{}_{2}/\mathrm{HCOO}{}^{-}}$ (CH_3_CN, H_2_O)=−1.32 V vs. Fc^+^/Fc.^[53]^ [b] FE${}_{\mathrm{HCOO}{}^{-}}$ and FE${}_{H{}_{2}}$ are mean values (*n*=2 or 3 replicates). [c] Energy efficiency=*E*_T_/*E*×FE${}_{\mathrm{HCOO}{}^{-}}$ , where *E* ^0^ ${}_{\mathrm{CO}{}_{2}/\mathrm{HCOO}{}^{-}}$ (CH_3_CN, H_2_O). [d] Total charge transferred 10 C. [e] The electrode used in this electrolysis was the same electrode used first in the entry 4 electrolysis under experimental conditions of entry 4, but in the absence of complex [1].

**Figure 5:**
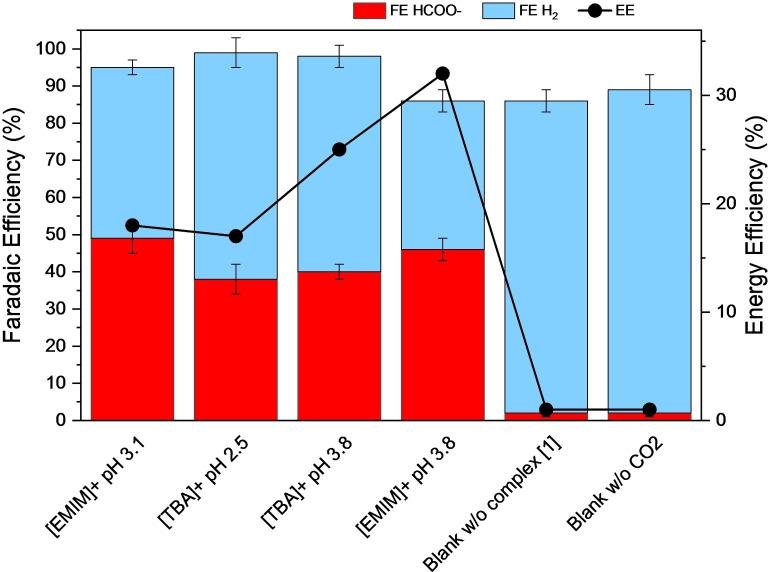
FE [%] and energy efficiency (EE) [%] electrolysis results obtained at −3.33 mA cm^−2^ in CO_2_‐saturated acidic aqueous solution using different electrolytes and proton source in an electrochemical two‐compartments H type cell.

Figure 5 and Table 3 show the CCE (applied current density −3.33 mA cm^−2^) results obtained with complex [1] in acidic aqueous CO_2_‐saturated solution using either [TBA][BF_4_] or [EMIM][BF_4_] as the electrolyte (see Figures S6 and S7). Different pH of the solution is reached in the acidic range between 2.5 and 3.8 depending on the particular electrolyte composition. Formate (p*K*_a_=3.75) in such acidic solutions exists under both protonated (HCOOH) and non‐protonated (HCOO^−^) forms. Remarkably, complex [1] allows significant CO_2_ conversion to formic acid/formate under these conditions, representing one of the rare examples of a homogeneous molecular complex performing CO_2_ conversion in acidic aqueous solution.

**Table 3:** Constant current electrolysis at −3.33 mA cm^−2^ in CO_2_‐saturated acidic aqueous solution using different electrolytes and proton source in an electrochemical two compartments H type cell. Total electrolysis duration to circulate 10 C.

Entry	Electrolyte	pH_0_	pH_f_	*E*_cat_^average^ [V] vs. Ag/AgCl	*η* [V]	FE${}_{\mathrm{HCOO}{}^{-}}$ ^[a]^ [%]	FE${}_{H{}_{2}}$ ^[a]^ [%]	Energy efficiency [%]
1	0.5 m [EMIM][BF_4_]	3.1	3.4	−1.6	1.02^[c]^	49±4	46±2	18
2	0.1 m [TBA][BF_4_]^[b]^+0.1 m CH_3_COOH	2.5	3.1	−1.2	0.65^[d]^	38±4	61±4	17
3	0.1 m [TBA][BF_4_]^[b]^+0.1 m CH_3_COO^−^/CH_3_COOH	3.8	3.8	−1.0	0.38^[e]^	40±2	58±3	25
4	0.1 m [EMIM][BF_4_]+0.1 m CH_3_COO/CH_3_COOH	3.8	3.9	−0.9	0.28^[e]^	46±3	40±3	32
5^[f]^	0.1 m [TBA][BF_4_]^[b]^+0.1 m CH_3_COO^−^/CH_3_COOH	3.8	3.8	−1.3	0.68^[e]^	2±1	84±3	1
6^[g]^	0.1 m [TBA][BF_4_]^[b]^+0.1 m CH_3_COO^−^/CH_3_COOH	3.8	3.7	−1.0	0.38^[e]^	2±1	87±4	1

[a] FE${}_{\mathrm{HCOO}{}^{-}}$ and FE${}_{H{}_{2}}$ are mean values (*n*=2 replicates). [b] 0.1 m is the maximum solubility of [TBA][BF_4_] in aqueous solution. [c] *E* ^0^ ${}_{\mathrm{CO}{}_{2}/\mathrm{HCOOH}}$ =−0.58 V vs. Ag/AgCl (pH=3.1). [d] *E* ^0^ ${}_{\mathrm{CO}{}_{2}/\mathrm{HCOOH}}$ =−0.55 V vs. Ag/AgCl (pH=2.5). [e] *E* ^0^ ${}_{\mathrm{CO}{}_{2}/\mathrm{HCOOH}}$ =−0.62 V vs. Ag/AgCl (pH=3.8). [f] No complex [1] in solution. [g] No CO_2_ in solution (Ar bubbling). pH_0_ and pH_f_ represent initial and final electrolysis solution pH, respectively. Energy efficiency=*E*_T_/*E*×FE${}_{\mathrm{HCOO}{}^{-}}$ , where *E*_T_=*E* ^0^ ${}_{\mathrm{CO}{}_{2}/\mathrm{HCOOH}}$ (H_2_O).

Initially, we evaluated the CO_2_RR performance in the presence of [EMIM][BF_4_] as sole electrolyte, which exhibits an acidic pH and a relevant FE${}_{\mathrm{HCOO}{}^{-}}$ of 49 % together with a 1 V overpotential (entry 1 in Table 3). A similar solution pH is achieved by combining [TBA][BF_4_] and acetic acid as proton donor source in solution (entry 2 in Table 3). This goes along with an evident diminution in overpotential (*η*=0.65 V), which is further improved at buffered pH of 3.8 by mixing acetic acid and acetate (*η*=0.38 V, entry 3 in Table 3). However, a significant decrease in selectivity towards formic acid/formate is also observed in both cases (Figure 5 and entries 2 and 3 in Table 3). Then, the substitution of [TBA[BF_4_] for [EMIM][BF_4_] induces a rise on the production of formic acid/formate (FE${}_{\mathrm{HCOO}{}^{-}}$ /FE${}_{H{}_{2}}$ ratio shifts from 0.7 to 1.2 by comparing entries 3 and 4 in Table 3, respectively). This effect cannot be ascribed to an increase of the concentration of CO_2_ in solution, although CO_2_ is more soluble in imidazolium‐based ILs than in aqueous solution, because the amount of IL present as electrolyte (less than 1 mol %) is not large enough to modify the CO_2_ concentration in solution. However, the presence of [EMIM][BF_4_] at the double layer could locally increase the molecular catalyst concentration at the electrode surface. In contrast, identical formate production was obtained in additional electrolysis performed by increasing the molecular catalyst concentration from 1 to 5 mm, which demonstrates that a higher concentration of molecular catalyst is not responsible for the formate production enhancement observed in the presence of ILs. Furthermore, the minimum overpotential required for reaching −3.33 mA cm^−2^ (*η*=0.28 V) is achieved by combining [EMIM][BF_4_] and acetic acid/acetate buffer in solution (entry 4 of Table 3). Comparing those results with previously reported molecular catalysts is not easy because experimental conditions vary from one study to another. In any case, 0.28 V seems to be among the lowest overpotential values reported so far for a homogeneous molecular catalyst producing formate in aqueous solution.[^14^, ^54^, ^55^] We only found in the literature two molecular complexes that behave similarly in terms of energy efficiency, an Ir pincer complex,^[54]^ which displays an overpotential of 0.8 V at −0.60 mA cm^−2^, but requires small amounts (≈1 %) of acetonitrile in solution, and an iron carbonyl cluster [Fe_4_N(CO)_12_]^−^,^[55]^ which displays 0.35 V at −4 mA cm^−2^. However, neutral aqueous solutions were used in both cases. Thus, as far as the authors are aware, not a single example of molecular catalyst for electrocatalytic formate production in such an acidic pH is reported in the literature so far. In addition to this, an interesting energy efficiency (32 %) (Figure 5), together with partial HER suppression in comparison with [TBA]^+^ are achieved (FE${}_{H{}_{2}}$ decreases from 58 to 40 % as comparing entries 3 and 4 in Table 3). Remarkably, this performance at low overpotential is stable in long term CCE (Figure S8). Furthermore, buffered acidic conditions limit pH changes during CCE, as shown in entries 3–6 of Table 3. In contrast, unbuffered solutions reported in entries 1 and 2 show an undesired, progressive solution alkalization during electrolysis. Figure 5 also shows control experiments without complex [1] (entry 5 in Table 3 and Figure S7) or without CO_2_ (entry 6 in Table 3 and Figure S7), which demonstrate the negligible effect of the electrode catalyzing direct CO_2_ conversion and confirm CO_2_ as the only source of carbon to generate formic acid/formate, respectively. In addition to this, the stability of the IL present in solution during electrolysis was demonstrated by ^1^H nuclear magnetic resonance (NMR) spectroscopy, since identical spectra of the IL in solution were obtained before and after the electrochemical reaction (Figure S9).

Finally, we performed DFT calculations to provide insights into the effects of the [EMIM]^+^ cation on the activity of complex [1]. Previous computational studies on the reduction of CO_2_ catalyzed by complex [1]^[50]^ showed that the [Rh^III^(bpy)(Cp*)H]^+^ intermediate tends to evolve towards the more stable [Rh^I^(bpy)(HCp*)]^+^ species bearing a protonated Cp* ligand (see Figure S10a).^[50]^ For this reason, we analyze the interactions between the [Rh^I^(bpy)(HCp*)]^+^ species and [EMIM]^+^. Notably, our calculations reveal that the formation of a π cation⋅⋅⋅π interaction between the catalyst and [EMIM]^+^ represented in Figure S10b stabilizes the lowest unoccupied molecular orbital (LUMO) of the [Rh^I^(bpy)(HCp*)]^+^ species facilitating its reduction. Accordingly, the calculated reduction potential is lowered by around 170 mV (Figure S10c), which is in fairly good agreement with the experimental shift (110 mV) observed in the *E*_cat_ value when comparing [TBA][PF_6_] and [EMIM][PF_6_] as supporting electrolyte (Table 1). As expected, the interaction between the complex and [EMIM]^+^ is further stabilized upon reduction of the bipyridine ligand (see Figure S10d).

**Figure 6:**
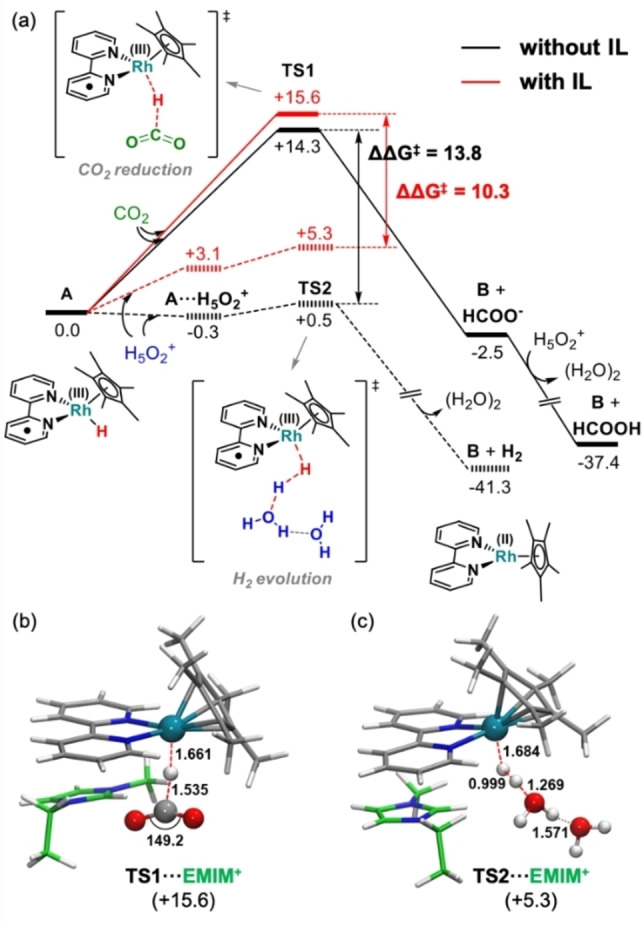
(a) Calculated Gibbs free energy profile [kcal mol^−1^] for the CO_2_‐reduction and the H_2_‐evolution reactions (solid and dashed lines, respectively) promoted by complex [1] in acetonitrile in the absence (black lines) or presence (red lines) of an explicit [EMIM]^+^ cation. (b, c) DFT‐optimized geometries for the transition states **TS1** and **TS2** in the presence of [EMIM]^+^, respectively. Main distances are shown in Å and relative free energies are given in parentheses in kcal mol^−1^.

Figure 6a compares the Gibbs free‐energy profiles in acetonitrile solution for formate and H_2_ production catalyzed by complex [1] in the presence or absence of an explicit [EMIM]^+^ interacting at the bipyridine ligand. Starting from the active form of the catalyst **A**, that is, the [Rh^III^(bpy⋅^−^)(Cp*)H] species (dashed frame in Scheme S1), the reduction of CO_2_ in the absence of IL takes place through **TS1** overcoming a free‐energy barrier of 14.3 kcal mol^−1^. This generates a formate ion, which is spontaneously protonated, and a Rh^II^ species **B**. The latter might undergo disproportionation to generate a Rh^I^ and a Rh^III^ species[^56^, ^57^] or be easily reduced back to Rh^I^ at the working onset potential. The HER in the absence of IL occurs through H−H coupling between **A** and a Zundel cation (H_5_O_2_^+^) (**TS2**) overcoming a very smooth energy barrier of 0.8 kcal mol^−1^ from a slightly stabilizing van der Waals adduct. The formation of the H_2_ product releasing a water dimer and species **B** is highly exergonic (>40 kcal mol^−1^). Note that although the standard‐state free‐energy barrier for HER is significantly lower than that for CO_2_RR, the experimental concentration of protons is expected to be several orders of magnitude lower than that of CO_2_, balancing the rate of both pathways and explaining the experimentally observed product distribution (entries 1 and 4 in Table 2). As shown in red lines in Figure 6a, the incorporation of [EMIM]^+^ at the electrode interface scarcely affects the free‐energy barrier for CO_2_RR, showing only a slight increase of 1.3 kcal mol^−1^ that lies within the limits of computational uncertainty. Conversely, the HER pathway is more significantly affected, showing an increase of 4.5 kcal mol^−1^ in the free‐energy barrier upon the incorporation of [EMIM]^+^. Notably, this reduces the free‐energy difference between **TS1** and **TS2** from 13.8 to 10.3 kcal mol^−1^ in acetonitrile when [EMIM]^+^ is present, thus shifting the product distribution in favor of formic acid/formate, which can qualitatively explain the experimental selectivity trend observed. Figure 6b and c display the transition states for CO_2_RR and HER in the presence of [EMIM]^+^. The stronger impact on the HER pathway can be ascribed to the cationic nature of the [Rh^III^(bpy⋅^−^)(Cp*)H]⋅⋅⋅[EMIM]^+^ complex. The latter might prevent to some extent the approach of other positively charged species such as a free protons, disfavoring the HER process via electrostatic repulsion. In fact, this can be already appreciated in going from species **A** to the **A**⋅⋅⋅**H_5_O_2_** ^**+**^ adduct, which becomes unfavorable when [EMIM]^+^ is attached to the catalyst structure (Figure 6, red dashed lines). Analogous results are also obtained in aqueous solution (reducing the free‐energy difference from 13.1 to 9.8 kcal mol^−1^). It is worth mentioning that having slightly higher free‐energy barriers for the hydride transfer step in the presence of [EMIM]^+^ together with a higher experimental current density may sound counterintuitive. However, one should note that the intensity of the catalytic curve might depend on the rate at which the catalyst is reduced at the electrode surface to generate its active species and not on the kinetics of the subsequent, thermally activated chemical step. Thus, bearing in mind the positive impact of [EMIM]^+^ in facilitating the reduction of the catalyst (Figure S10c), the observed faster electron transfer kinetics in the presence of [EMIM]^+^ is the expected outcome.

Interestingly, a Re^I^ complex catalyzing CO_2_ conversion to CO[^43^, ^44^] also exhibits π^+^–π interactions with [EMIM]^+^ and shares bypiridine with complex [1] as a common ligand. Then, it is highly probable that other active molecular catalysts for CO_2_RR containing bipyridine ligands will exhibit a significant promoting effect by incorporating imidazolium‐based ILs at the electrode/solution interface.

## Conclusion

Using a Rh‐based model molecular catalyst in solution for CO_2_ conversion to formic acid/formate (complex [1]), we demonstrate the significant impact of tuning the electrical double layer by electrolyte engineering with ionic liquids (ILs) on both the catalytic activity and selectivity. Firstly, the presence of imidazolium‐based ILs was found to decrease the overpotential both in acetonitrile and acidic aqueous solution. Density functional theory (DFT) calculations suggested the formation of π^+^–π interactions between the catalyst and [EMIM]^+^. The latter facilitate the reduction of the catalyst to generate its active form, explaining thus the decrease in overpotential. Secondly, [EMIM]^+^ cations were found to play a key role partially inhibiting the hydrogen evolution side reaction via electrostatic repulsion between [EMIM]^+^ and free protons, which significantly improves the selectivity of the CO_2_ reduction reaction (CO_2_RR) to formic acid/formate production. Any potential hydrophobic effect provided by the presence of imidazolium‐ILs at the electrode–electrolyte interface was ruled out, since the enhancement observed in the faradaic efficiency of HCOO^−^ (FE${}_{\mathrm{HCOO}{}^{-}}$ ) was not accompanied by any drop in the current density, which actually increased (Figures 3 and 4). This is indeed in contrast with the effect of adding long‐chain cationic surfactants in solution, which provokes a significant drop in current density.[^35^, ^37^] Therefore, complex [1] in the presence of [EMIM]^+^ in acetonitrile exhibits a FE${}_{\mathrm{HCOO}{}^{-}}$ ≥90 % and a maximal energy efficiency for CO_2_ conversion to formate of 66 %, thus placing complex [1] among the top‐performing molecular catalysts reported in the literature.[^14^, ^54^, ^55^]

The IL‐dependent partial inhibition effect on the hydrogen evolution reaction also allowed, for the first time, efficient CO_2_RR catalyzed by a molecular catalyst under acidic aqueous conditions. A remarkable energy efficiency of 32 % was achieved with complex [1] in acetate buffered solution, thanks to a FE${}_{\mathrm{HCOO}{}^{-}}$ of around 45 % coupled with an overpotential of 0.28 V for achieving 3.3 mA cm^−2^, one of the lowest overpotential values reported thus far.^[14]^ Overall, these results in acidic aqueous solution are very promising in order to improve the carbon balance in CO_2_RR by limiting CO_2_ losses due to carbonate and bicarbonate generation, which commonly happens in alkaline and neutral aqueous solutions.

## Experimental Section

### Reactants

Anhydrous acetonitrile (CH_3_CN, 99.99 %), sodium acetate (CH_3_COONa ⋅ 3H_2_O, >99 %), tetrabutyl ammonium hexafluorophosphate ([TBA][PF_6_], >99 %), tetrabutyl ammonium tetrafluoroborate ([TBA][BF_4_], >99 %), and 2,2′‐bipyridyl (≥99 %) were all purchased from Sigma‐Aldrich. Acetic acid (CH_3_COOH, >99.5 %) was purchased from TCI chemicals. Complex [1] precursor dichloro(pentamethylcyclopentadienyl)rhodium(III) dimer [Rh(Cp*)Cl_2_]_2_ (99 %) was purchased from Strem Chemicals. ILs: 1‐ethyl‐3‐methylimidazolium hexafluorophosphate ([ΕΜΙΜ][PF_6_]) (99 %), 1‐ethyl‐3‐methylimidazolium tetrafluoroborate ([ΕΜΙΜ][BF_4_]) (>98 %), 1‐butyl‐3‐methylimidazolium hexafluorophosphate ([ΒΜΙΜ][PF_6_]) (99 %), and 1‐butyl‐1‐methylpyrrolidinium hexafluorophosphate ([ΒΜΡyrr][PF_6_]) (99 %) were all purchased from Io‐li‐tec (Germany). Ferrocene (98 %) was purchased from Merck. All reactants were used without any further purification. All aqueous solutions were prepared with ultrapure water (18.2 MΩ cm, Millipore).

### Synthesis of complex [1]

The following synthesis was adapted from existing protocols in the literature.[^58^, ^59^] A methanol solution (30 mL) of 1 equiv. [Rh(Cp*)Cl_2_]_2_ (200 mg, 0.32 mmol) and 2 equiv. 2,2’‐bipyridine (120 mg, 0.76 mmol) was stirred at RT for 2 h in the dark. The resulting clear orange–yellow solution was evaporated until dry. The yellow solid was dissolved in a minimal quantity of acetonitrile and precipitated upon the addition of ethyl acetate, then collected on a Buchner funnel and dried under vacuum. The purity of the final precipitate was verified by ^1^H NMR spectroscopy according to the literature.^[58]^ Figure S11 shows the ^1^H NMR spectrum of [Rh(bpy)(Cp*)Cl] (300 MHz, CD_3_CN): *δ*=1.61 (s, 15H), 7.71 (ψt, *J*=7.2 Hz, 2H), 8.13 (dt, *J*=7.7 Hz, 2H), 8.30 (d, *J*=8.0 Hz, 2H), 8.78 ppm (d, *J*=5.5 Hz, 2H).

### Electrochemical studies

All electrochemical experiments were performed either on SP‐300 or VSP‐300 potentiostats/galvanostats (Bio‐Logic Science Instruments SAS) and were conducted at RT (20±2 °C) in different solvents (CH_3_CN, H_2_O, and mixtures of both of them). Either [TBA][PF_6_], [TBA][BF_4_], or one of the aforementioned ILs was used as a supporting electrolyte in solution (0.1–0.5 m). In some cases, 0.1 m CH_3_COO^−^/CH_3_COOH buffer solution (pH=3.8) was used as additional supporting electrolyte in aqueous solution. Ar (>99.99 %) and CO_2_ (>99.99 %) gases used to saturate solutions were purchased from Air Liquide. The cyclic voltammetry (CV) experiments were carried out in a three‐electrodes setup, with a 3 mm diameter GC disc electrode (0.07 cm^2^) as a working electrode (BioLogic), which was polished on a polishing cloth on a 1 μm diamond suspension (Struers), sonicated for 10 s in water, and dried prior to experiments. A platinum wire was used as a counter electrode (diameter=0.5 mm, Alfa Aesar, 99.5 % purity) and was previously flame annealed. The reference electrode used in all cases was a conventional Ag/AgCl/KCl_sat_ reference electrode (BioLogic) separated from the solution by a salt bridge. In acetonitrile however, all potentials were calibrated using the ferrocenium/ferrocene (Fc^+^/Fc) redox couple as an internal standard, which was added in the solution at the end of each experiment. CVs were run at 0.01 V s^−1^ scan rate and only the third steady state cycle of all CVs is shown, unless otherwise stated in the text.

Catalytic response (*j*_cat_/*j*_p_) from CV was calculated as the ratio between the highest value of reduction peak current density exhibited under catalytic conditions (CO_2_) (*j*_cat_) and the highest value of reduction peak current density exhibited under inert conditions (Ar) (*j*_p_). Catalytic potential (*E*_cat_) corresponds to the value at the maximum of the catalytic current density and (*E*_cat/2_) corresponds to the half wave catalytic potential.

A gastight two‐compartment electrochemical H‐type glass cell with a glass frit separating anolyte (5 mL) and catholyte (10 mL) solutions was used in all electrolyses reported here. Controlled potential or current electrolysis (CPE and CCE, respectively) were performed in acetonitrile solution containing 5 % *v*/*v* H_2_O and 0.1–0.5 m of supporting electrolyte previously saturated with CO_2_ by gas bubbling in both catholyte and anolyte, but no continuous CO_2_ gas was purged during the electrolysis. 1 mm of complex [1] was only added in the catholyte. The working electrode was a 1 cm^2^ GC plate (1 mm thick, type 2, from Alfa Aesar) the counter electrode was a 5 cm^2^ GC rod (Alfa Aesar) and a conventional Ag/AgCl/KCl_sat_ electrode separated from the solution by a salt bridge, which was calibrated with ferrocene as an internal redox reference, was used as a reference electrode. Ohmic losses in the cell were minimized by achieving the minimal distance between electrodes and keeping magnetic stirring during the electrolysis. Additionally, 85 % of the ohmic drop was compensated by the *iR* correction module of the potentiostat. CCE were conducted either in acetonitrile solution containing between 5 % and 50 % *v*/*v* H_2_O or in purely aqueous solutions. However, only [TBA][BF_4_] and [ΕΜΙΜ][BF_4_] were soluble in aqueous solution among all the electrolytes studied here. A 1 cm^2^ GC plate was used as working electrode when acetonitrile solutions were electrolyzed. In contrast, a 3‐dimensional reticulated vitreous carbon (RVC) foam (geometrical area=3 cm^2^) was used as working electrode when aqueous solutions were electrolyzed {rectangular 3‐dimensional Duocel® RVC foam [pores per inch (PPI)=45 and *l*×*w*×*h*=1.5×0.5×2 cm^3^] from ERG Materials and Aerospace Co.}. In all cases, the current density was calculated using the electrode geometrical area. All CPE and CCE experiments were performed with 2 or 3 replicates to check results reproducibility.

### Analytical quantification of products

Gas products were quantified by gas chromatography (Model 8610 C SRI Instruments) equipped with thermal conductivity detector (TCD) and flame ionization detector (FID) from 50 μL aliquots of the headspace of both compartments. Only hydrogen (H_2_) was detected as a gas product. Liquid products were evaluated using an ionic exchange chromatograph (IC) (Metrohm 883 Basic IC) equipped with a Metrosep A Supp 5 column and a conductivity detector. Only formate was detected. A typical quantification of formate by IC required the sampling of 50 μL of solution from catholyte and/or anolyte, followed by a (200–400) dilution in ultrapure water and a final injection of 20 μL into the IC chromatograph. FE of each reaction product is calculated from the ratio between the charge consumed to form each product and the total circulated charge.^[60]^ However, the total circulated charge is corrected to discount the initial three electrons consumed by complex [1] (1 mm in solution) necessary to generate its active form. Catalyst activation charge=[number of electrons×Faraday constant×mol of catalyst]=[3×96485×6.06×10^−6^]=1.82 C. In order to compare all CPE and CCE results, a constant total charge (15 C in acetonitrile solutions and 10 C in aqueous solutions) has been used in all electrolysis. The overpotential (*η*) was calculated from the difference between the electrolysis applied potential and *E* ^0^ ${}_{\mathrm{CO}{}_{2}/\mathrm{HCOO}{}^{-}}$ (CH_3_CN, H_2_O)=−1.32 V vs. Fc^+^/Fc or *E* ^0^ ${}_{\mathrm{CO}{}_{2}/\mathrm{HCOOH}}$ (H_2_O)=−0.199 V vs. standard hydrogen electrode (SHE) in acetonitrile^[53]^ and aqueous solutions,^[61]^ respectively. Additionally, *E* ^0^ ${}_{\mathrm{CO}{}_{2}/\mathrm{HCOOH}}$ in aqueous solution was transferred from SHE to the Ag/AgCl/KCl_sat_ reference electrode taking into account the solution pH and using the following Equation 1:

(1) $$E_{CO_{2}/HCOOH}^{0}\left(Ag/AgCl\right)=E_{CO_{2}/HCOOH}^{0}\left(SHE\right)-0.059\;pH-0.197$$

The cathodic half reaction energy efficiency (EE) was calculated for CO_2_ conversion to formate reaction according to the following Equation 2:^[19]^

(2) $$EE\;\left[\%\right]=\frac{E_{T}}{E}\times {FE}_{HCOO^{-}}$$

where *E*_T_ is the thermodynamic potential in volts required for the electrocatalytic reduction of CO_2_ to formate, whereas *E* and FE${}_{\mathrm{HCOO}{}^{-}}$ represent the experimental cathode potential applied in volts and the formate FE [%], respectively.

### Computational methods

DFT calculations were carried out at the ωB97X−D level^[62]^ using the Gaussian 16 (rev. C.01) quantum chemistry software.^[63]^ The LANL2DZ^[64]^ basis set and associated pseudopotentials were used to describe Rh ions, which were supplemented by one shell of f‐type polarization functions.^[65]^ Remaining atoms were treated with the Pople‐type 6‐31G(d,p) basis set.[^66^, ^67^, ^68^] Solvent effects of acetonitrile and water were included in the geometry optimizations and energy calculations by means of the IEF‐PCM implicit solvent model,^[69]^ as implemented in Gaussian 16. The nature of the stationary points on the potential energy surface was confirmed via normal‐mode analysis calculations. The standard‐state correction to switch from the reference state of 1 atm used in the Gaussian code to 1.0 m in solution at 25 °C (+1.89 kcal mol^−1^) was applied to the free energy of all the species. A dataset collection of the computational results is available in the ioChem‐BD repository^[70]^ and can be accessed via: DOI: 10.19061/iochem‐bd‐6‐109.

## Conflict of interest

The authors declare no conflict of interest.

1

## Supporting information

As a service to our authors and readers, this journal provides supporting information supplied by the authors. Such materials are peer reviewed and may be re‐organized for online delivery, but are not copy‐edited or typeset. Technical support issues arising from supporting information (other than missing files) should be addressed to the authors.

Supporting Information

## Data Availability

The data that support the findings of this study are available from the corresponding author upon reasonable request.
