# Supplementary material for: Electrocatalytic Conversion of CO2 to Formate at Low Overpotential by Electrolyte Engineering in Model Molecular Catalysis
Source: ChemSusChem. 2022 Nov 11;15(24):e202201566. doi: 10.1002/cssc.202201566 (PMC10100316; doi:10.1002/cssc.202201566)
Supplement: Supplementary file 1 — Supporting Information [file CSSC-15-0-s001.pdf]

ChemSusChem

Supporting Information

# Electrocatalytic Conversion of $CO_2$ to Formate at Low Overpotential by Electrolyte Engineering in Model Molecular Catalysis

Elli Vichou, Albert Solé-Daura, Caroline Mellot-Draznieks, Yun Li, Maria Gomez-Mingot,* Marc Fontecave,* and Carlos M. Sánchez-Sánchez*© 2022 The Authors. ChemSusChem published by Wiley-VCH GmbH. This is an open access article under the terms of the Creative Commons Attribution License, which permits use, distribution and reproduction in any medium, provided the original work is properly cited.

# *Table of Contents*

*Scheme S1. Electrocatalytic $CO_2$RR and HER cycles of complex [1].* S3

*Figure S1. Cyclic voltammograms of complex [1] and different supporting electrolytes in acetonitrile solution under inert conditions .* S4

*Figure S2. Cyclic voltammograms of complex [1] and 0.5 M of different supporting electrolytes in acetonitrile solution under Ar: [TBA][PF6] and [EMIM][PF6].* S5

*Figure S3. Cyclic voltammogram of complex [1] in an acetonitrile/water 50/50 v/v solution containing [TBA][BF4] under $CO_2$.* S6

*Figure S4. Cyclic voltammograms of complex [1] in purely aqueous solution containing [TBA][BF4] or [EMIM][BF4] under Argon/$CO_2$.* S7

*Figure S5. Constant current electrolysis (CCE) of complex [1] in acetonitrile solution under $CO_2$ in presence of 5 % (vol.) $H_2O$ and different supporting electrolytes.* S8

*Figure S6. Constant current electrolysis (CCE) of complex [1] in acidic aqueous solution under $CO_2$ in presence of different supporting electrolytes.* S9

*Figure S7. Constant current electrolysis (CCE). Control experiments.* S10

*Figure S8. Long term constant current electrolysis (CCE) of complex [1] in aqueous solution under $CO_2$ in presence of [TBA][BF4] and acetate buffer solution.* S11

*Figure S9. Proton NMR of Entry 1 Table 3 (1mM complex 1 and 0.5 M EMIMBF$_4$ in $H_2O$ under $CO_2$) before (red plot) and after CCE (blue plot).* S12

*Figure S10. a) Proton/hydride tautomerism in the protonated form of the catalyst. b) Optimized geometry for the non-bonding interaction between activated complex [1] and [EMIM]$^+$. c) Schematic frontier MO diagram. d) Scheme on the reaction steps.* S13

*Figure S11. Proton NMR of complex [1]* S14

*References* S15

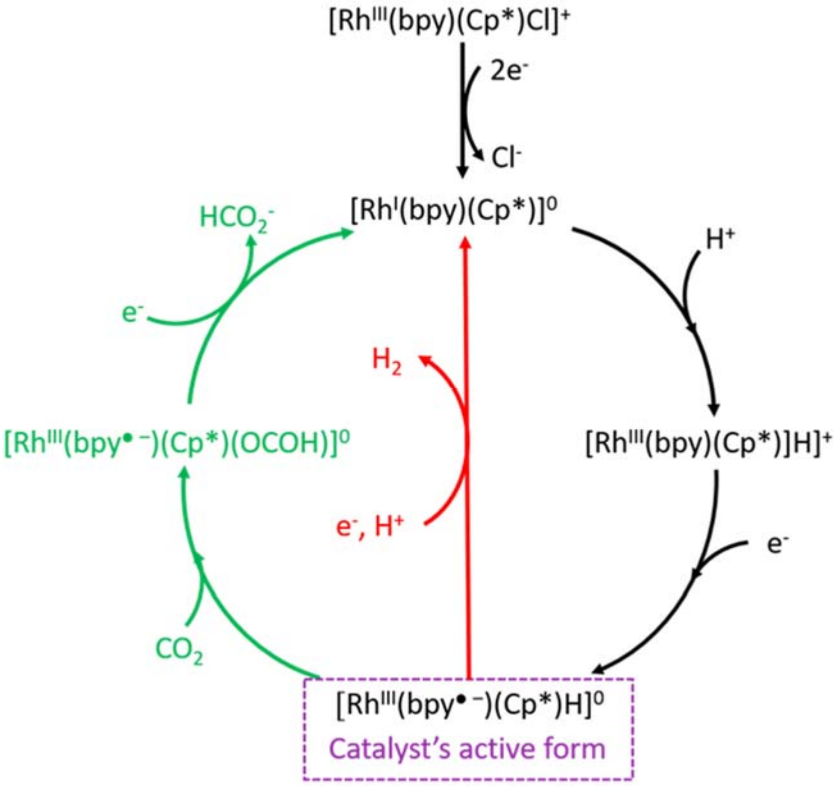


**Scheme S1**. Electrocatalytic cycle of complex [1] for either $CO_2$ to formate conversion (green pathway) or $H_2$ production (red pathway)[1,2]

**Figure S1a** compares the benchmark electrolyte cation $[TBA]^+$ and the pyrrolidinium cation $[BMPyrr]^+$, by keeping $[PF_6]^-$ as the counter anion in both cases. **Figure S1b** compares the benchmark electrolyte cation $[TBA]^+$ and three different imidazolium-based ILs, comprising two different cations ($[EMIM]^+$ and $[BMIM]^+$) and two different counter anions ($[PF_6]^-$ and $[BF_4]^-$).

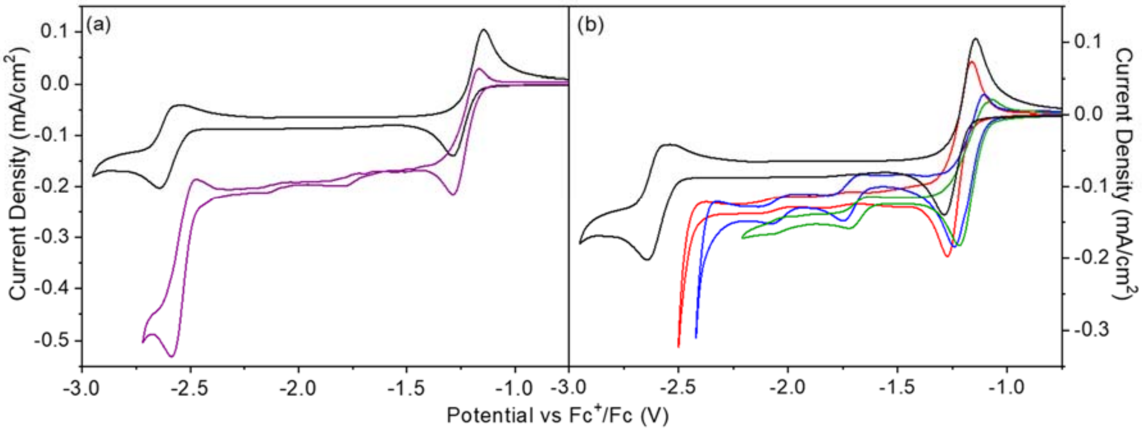


**Figure S1.** Cyclic voltammograms on GC electrode of 1 mM complex [1] and 0.5 M of different supporting electrolytes in acetonitrile solution under Ar. (a) $[TBA][PF_6]$ (black plot) and $[BMPyrr][PF_6]$ (purple plot) and (b) $[TBA][PF_6]$ (black plot), $[EMIM][PF_6]$ (red plot) and $[BMIM][PF_6]$ (blue plot) and $[EMIM][BF_4]$ (green plot). Scan rate 0.01 V $s^{-1}$.

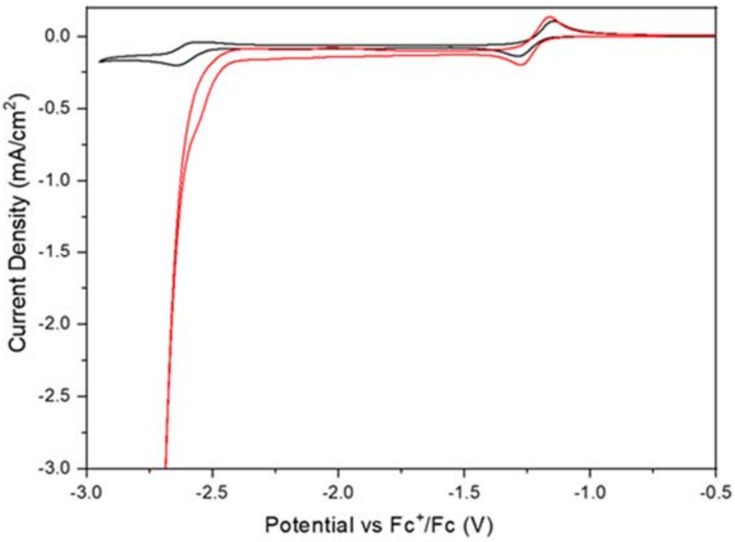


**Figure S2.** Cyclic voltammograms on GC electrode of 1 mM complex [1] and 0.5 M of different supporting electrolytes in acetonitrile solution under Ar. (a) [TBA][$PF_6$] (black plot) and [EMIM][$PF_6$] (red plot). Scan rate 0.01 V $s^{-1}$.

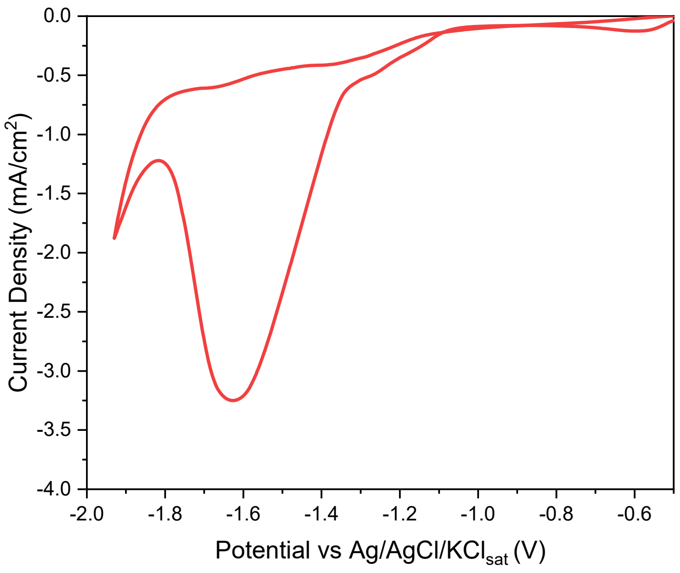


**Figure S3.** Cyclic voltammogram on GC electrode of 1 mM complex [1] in an acetonitrile/water 50/50 v/v solution containing 0.1 M of [TBA][$BF_4$] under $CO_2$. Scan rate 0.01 V $s^{-1}$.

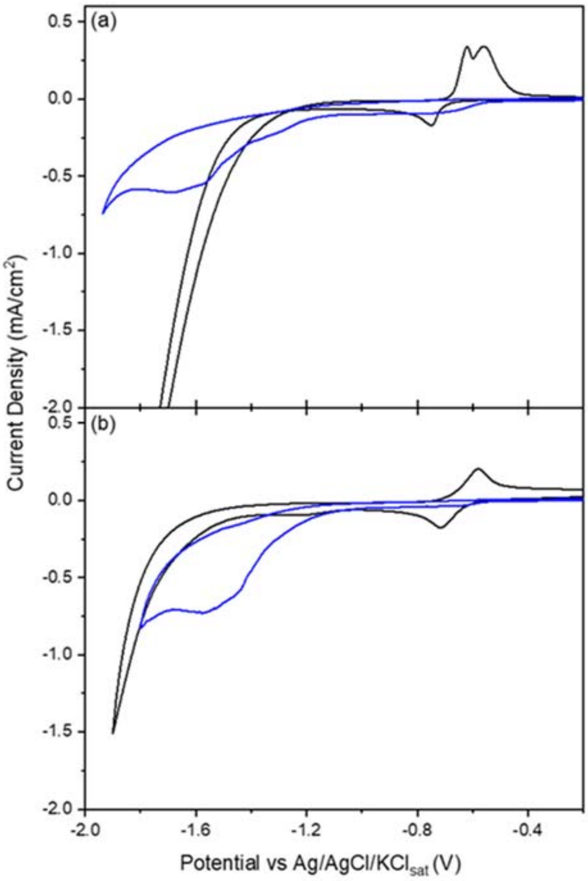


**Figure S4.** Cyclic voltammograms on GC electrode of 1 mM complex [1] in purely aqueous solution containing: (a) 0.1 M [TBA][$BF_4$] under Argon (black plot) or under $CO_2$ (blue plot) and (b) 0.5 M [EMIM][$BF_4$] under Argon (black plot) or under $CO_2$ (blue plot). Scan rate = 0.01 V $s^{-1}$.

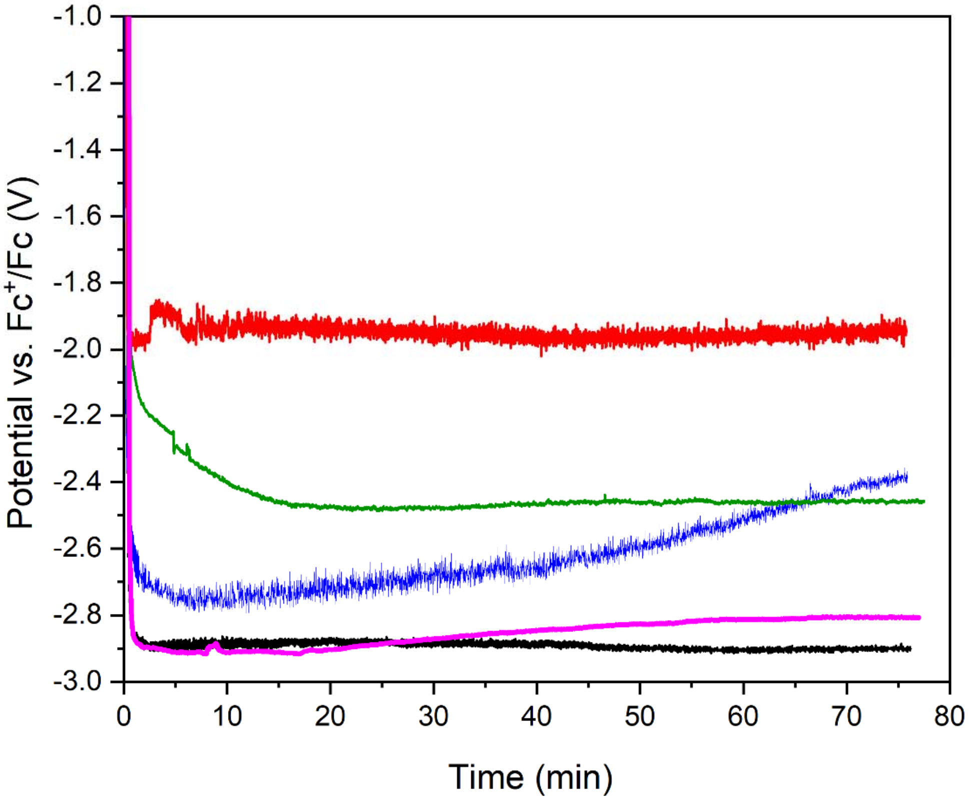


**Figure S5.** Constant current electrolysis (CCE) at -3.33 mA/cm$^2$ of 1 mM complex [1] in acetonitrile solution under $CO_2$ in presence of: 5 % (vol.) $H_2O$ and 0.5 M [TBA][$PF_6$] (black plot), 5 % (vol.) $H_2O$ and 0.5 M [TBA][$BF_4$] (magenta plot) 5 % (vol.) $H_2O$ and 0.5 M [EMIM][$PF_6$] (red plot) and 50 % (vol.) $H_2O$ with 0.5 M [TBA][$BF_4$] (blue plot). Blank CCE at -3.33 mA/cm$^2$ in the absence of complex [1] under $CO_2$ in presence of: 5 % (vol.) $H_2O$ and 0.5 M [TBA][$PF_6$] (green plot). Room temperature: 20 ± 2 °C, stirring rate: 400 rpm.

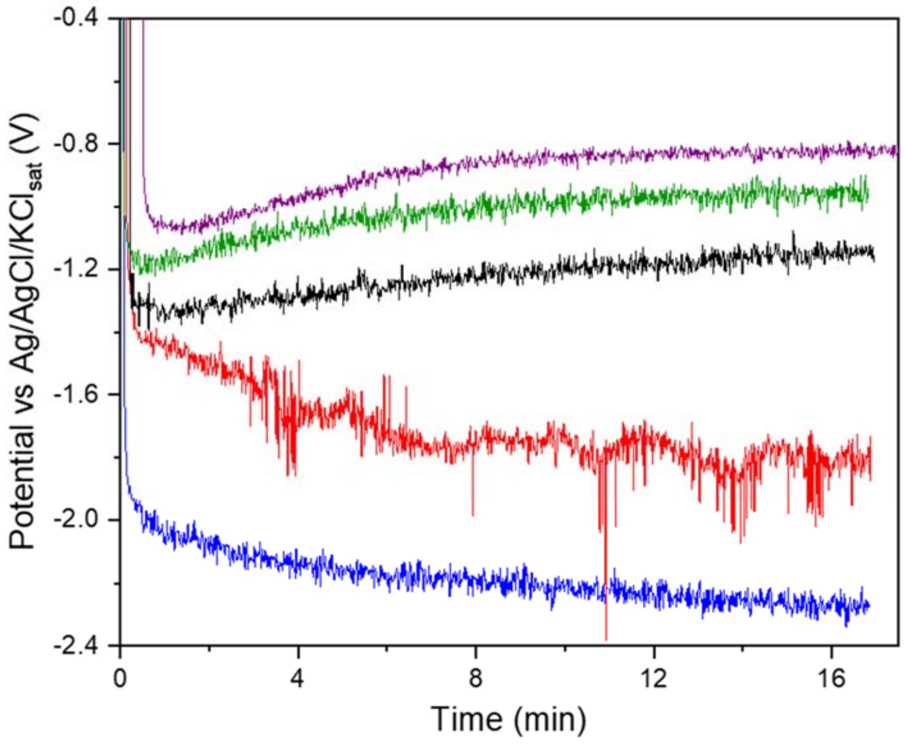


**Figure S6**. Constant current electrolysis (CCE) at -3.33 mA cm$^{-2}$ of 1 mM complex [1] in acidic aqueous solution under $CO_2$ in presence of: 0.1 M [TBA][$BF_4$] (blue plot), 0.5 M [EMIM][$BF_4$] (red plot), 0.1 M [TBA][$BF_4$] and 0.1 M acetic acid (pH=2.5) (black plot), 0.1 M [TBA][$BF_4$] and 0.1 M acetate buffer (pH = 3.8) (green plot) and 0.1 M [EMIM][$BF_4$] and 0.1 M acetate buffer (pH = 3.8) (purple plot). Room temperature: 20 ± 2 °C, stirring rate: 400 rpm.

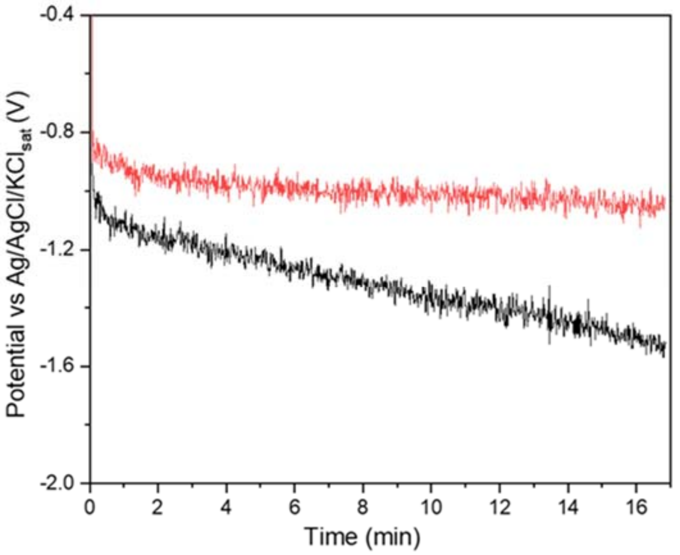


**Figure S7**. Control experiments: Constant current electrolysis (CCE) at -3.33 mA cm$^{-2}$ in 0.1 M [TBA][$BF_4$] + 0.1 M acetate buffer (pH = 3.8) aqueous solution in the presence of only $CO_2$ (black plot) or 1 mM complex [1] and Ar (red plot). Room temperature: 20 ± 2 °C, stirring rate: 400 rpm

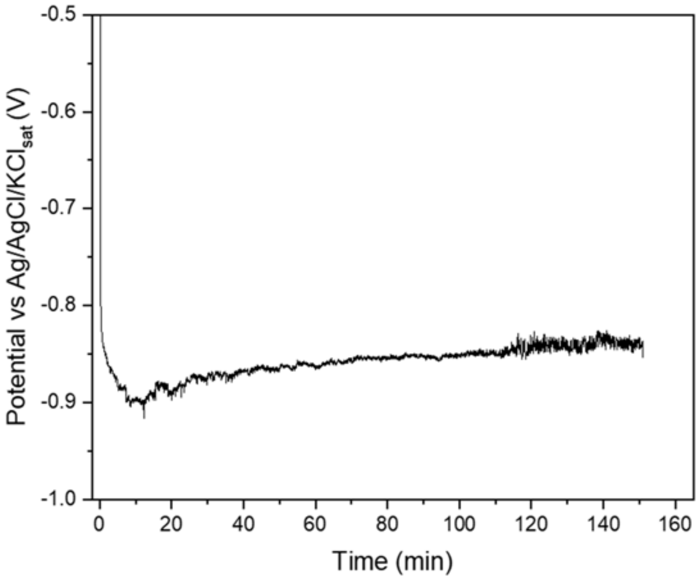


**Figure S8.** Long term constant current electrolysis (CCE) at -3.33 mA cm$^{-2}$ of 1 mM complex [1] in aqueous solution under $CO_2$ in presence of: 0.1 M [TBA][$BF_4$] + 0.1 M acetate buffer (pH = 3.8). Room temperature: 20 ± 2 °C, stirring rate: 400 rpm

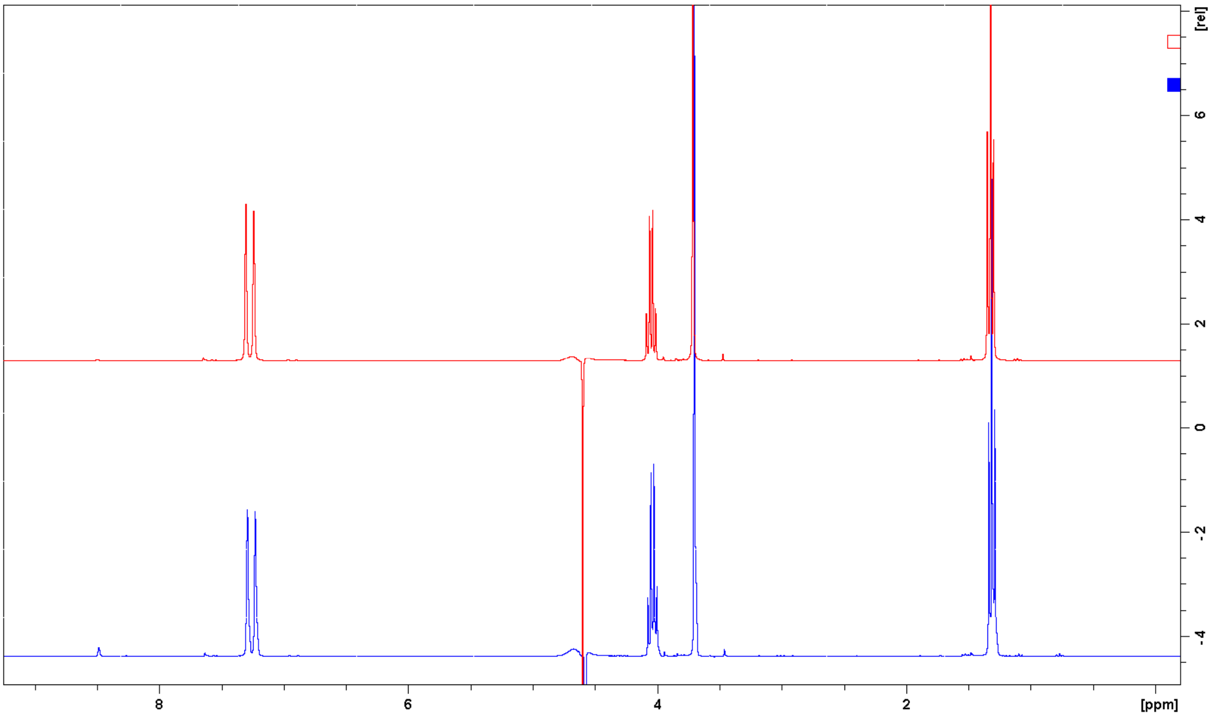


**Figure S9**. $^{1}$H NMR (300 MHz, $D_2O$) of Entry 1 in Table 3 (1 mM complex [1] and 0.5 M $EMIMBF_4$ in $H_2O$ under $CO_2$) before (red plot) and after CCE (blue plot) for a total charge of 10 C.

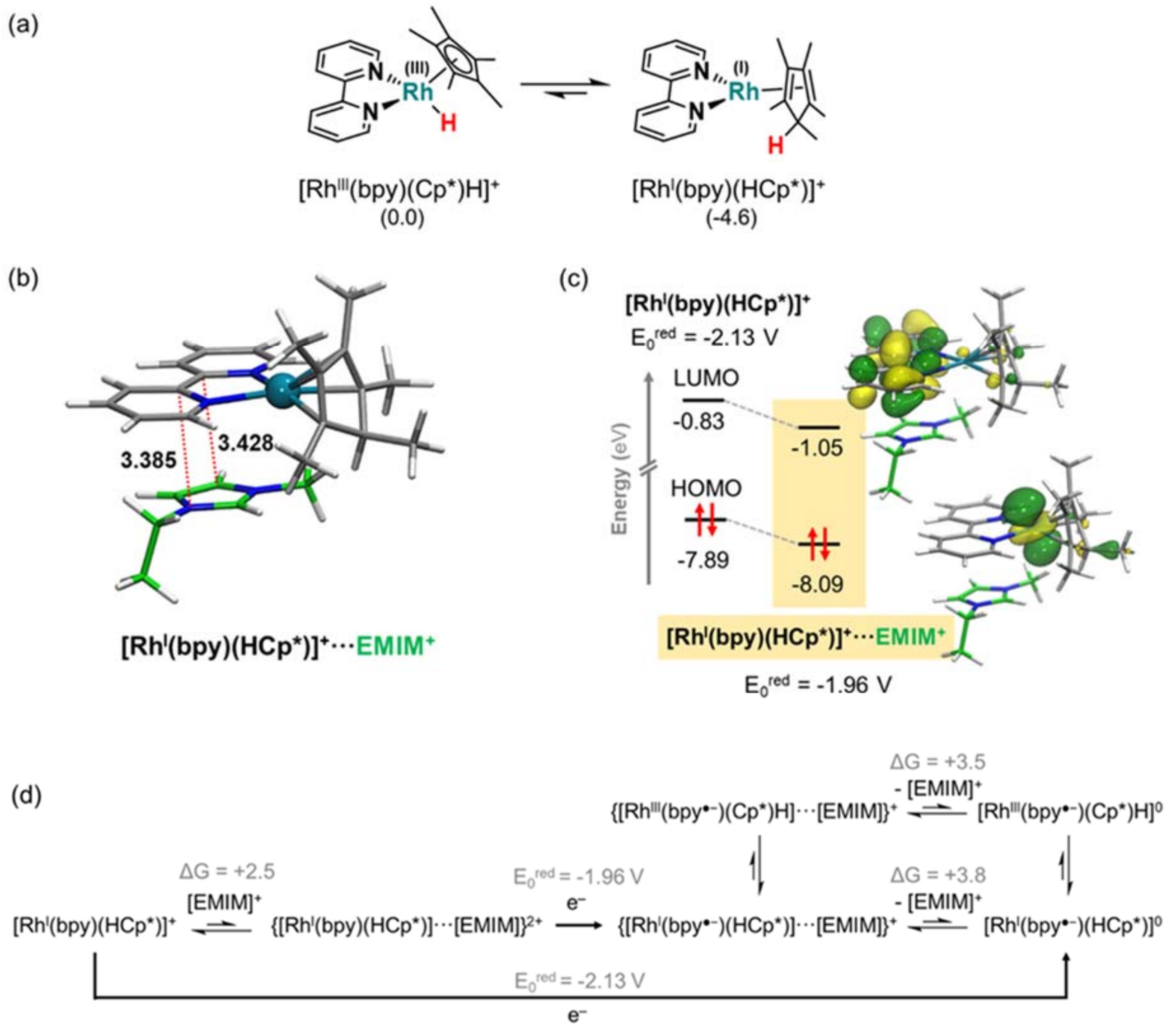


**Figure S10.** a) Proton/hydride tautomerism in the protonated form of the catalyst. Relative Gibbs free energies in parenthesis are given in kcal mol$^{-1}$. b) Optimized geometry for the non-bonding interaction between the [Rh$^{I}$(bpy)(HCp*)]$^{+}$ catalyst and [EMIM]$^{+}$. Key distances are shown in Å and carbon atoms of [EMIM]$^{+}$ are colored in green for clarity. c) Schematic frontier MO diagram showing the impact of the interaction with [EMIM]$^{+}$ on the energy levels and reduction potential of the [Rh$^{I}$(bpy)(HCp*)]$^{+}$ species. Orbital energies computed at the ωB97X-D level are given in eV and reduction potentials are referred to the Fc$^{+}$/Fc reference electrode. d) Possible relevant equilibria established in solution before and after the third reduction of the catalyst and in the presence of [EMIM]$^{+}$ cations. Gibbs free energies are given in kcal mol$^{-1}$. Reduction potentials are referred to the Fc$^{+}$/Fc reference electrode.

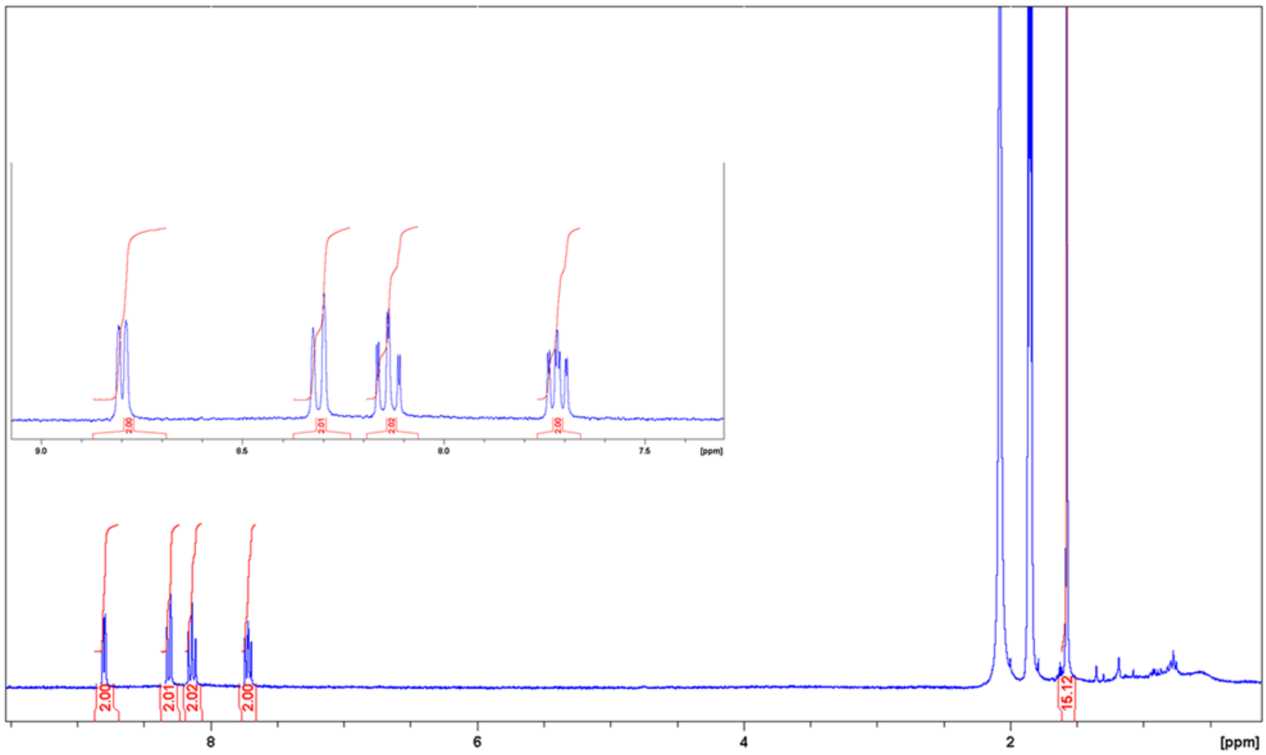


**Figure S11.** $^{1}H$ NMR (300 MHz, $CD_3CN$) of synthesized [Rh(bpy)(Cp*)Cl]: δ/ppm, 1.61 (s, 15H), 7.71 (ψt, J = 7.2 Hz, 2H), 8.13 (dt, J = 7.7 Hz, 2H), 8.30 (d, J = 8.0 Hz, 2H), 8.78 (d, J = 5.5 Hz, 2H).

## REFERENCES


[1] C. Caix, S. Chardon-Noblat, A. Deronzier, *J. Electroanal. Chem.* **1997**, *434*, 163–170.

[2] C. Caix, S. Chardon-Noblat, A. Deronzier, J.-C. Moutet, S. Tingry, *J. Organomet. Chem.* **1997**, *540*, 105–111.
